# The Gratitude Resentment and Appreciation Test-Revised Short (GRAT-RS): A Multidimensional Item Response Theory Analysis in Italian Workers

**DOI:** 10.3390/ijerph192416786

**Published:** 2022-12-14

**Authors:** Andrea Svicher, Letizia Palazzeschi, Alessio Gori, Annamaria Di Fabio

**Affiliations:** 1 Department of Education, Languages, Intercultures, Literatures and Psychology, (Psychology Section), University of Florence, 50135 Florence, Italy; 2 Department of Health Sciences (Psychology Section), University of Florence, 50135 Florence, Italy

**Keywords:** GRAT-RS, Gratitude Resentment and Appreciation Test-Revised Short, gratitude, multidimensional item response theory, healthy organizations, psychometric properties, strength-based prevention perspectives, workers

## Abstract

Gratitude is a promising resource from a healthy organizational perspective. It is related to many positive outcomes at work. The Gratitude Resentment and Appreciation Test-Revised Short (GRAT-RS) is the most widely used self-report questionnaire to detect gratitude. The present study examined GRAT-RS (the Italian version) by implementing multidimensional item response theory (MIRT) analyses to explore its psychometric properties. The participants were 537 Italian workers. Confirmatory factor analyses (CFA) of the GRAT-RS and MIRT analyses using the Grade Response Model were run. The MIRT discrimination and MIRT difficulty parameters were calculated. A test information function (TIF) and measure of reliability associated with (TIF) scores were also implemented. CFA highlighted that a bifactor model showed the best fit. Hence, MIRT analyses were carried out by implementing a bifactor model. The MIRT bifactor structure showed a good data fit with discrimination parameters ranging from good to excellent and adequate reliability. The good psychometric properties of GRAT-RS were confirmed, highlighting the questionnaire as a reliable tool to measure gratitude in Italian workers.

## 1. Introduction

The word “gratitude” stems from the Latin term “gratia”, which means “grace” [1]. This Latin root refers to generosity, kindness, gift, and the associated positive feelings involved in obtaining or receiving something for nothing [2]. For Lazarus [3], gratitude is an emotion that involves empathy. Indeed, people are able to feel gratitude when they recognize and appreciate what another individual has done for them [4]. In this line, McCullough, Emmons, and Tsang [5] consider gratitude as a disposition of individuals, defining it as “a generalized tendency to recognize and respond with grateful emotion to the roles of other people’s benevolence in the positive experiences and outcomes that one obtains” (p. 112). Thus, gratitude also regards the thankfulness for all the positive aspects of individual existence [1,6].

In brief, gratitude encompasses both cognitive and emotional processes. Cognitive ones refer to the following two phases [7]: first, judging whether a favorable outcome linked to feelings of happiness has occurred; second, attributing the feelings of happiness to external sources. Therefore, the emotional aspects of gratitude are inherent in the propensity to answer with a grateful emotion to the benevolence of others [5,8].

In the positive psychology framework [9,10], gratitude is recognized as a pillar, being in positive relation with well-being and psychological strengths [5,11]. Furthermore, gratitude is positively associated with a wide array of positive psychological outcomes, such as hope and optimism, and negatively related to variables of psychological distress, namely, depression, anxiety, and stress [5]. In a positive psychology background, gratitude was also found to contribute to psychological well-being in addition to the personality traits of the Big Five [6]. Gratitude is a variable that can be improved through training, differently from personality traits that are substantially stable [6]. Indeed, the literature showed a variety of specific training developed to improve gratitude and in a broader way well-being [12,13]. Accordingly, gratitude is a promising variable for the strength-based prevention perspectives [14] and for the primary prevention perspectives [15]. In this line, gratitude is considered a psychological strength to be increased to enhance well-being, applying specific and early training in different settings [16,17].

In organizations, gratitude is considered crucial for efficiency and productivity and, at the same time, was found to be related to organizational citizenship behaviors, prosocial organizational behaviors and the organizational climate [18]. Gratitude is therefore noteworthy in order to increase positive relationships, social support and well-being of workers, reduce negative emotions in the workplace, and improve the health and success of organizations [18]. Gratitude is also connected to meaningful work [19], underlining the possible value of gratitude in the processes relating to the passage from the meaning of work to meaningful life, expanding decent work [20,21].

It is possible to trace new research and intervention perspectives on gratitude, specifically in organizational contexts [22]. Gratitude appears to be involved in building positive relationships, a crucial aspect of healthy organizations [23] and healthier societies. Developing gratitude is a promising area to improve positive relationships and promote new aspects of relatedness, for example, in terms of positive management, organizational justice and leadership styles encompassing gratitude [24,25], including new forms of innovation in organizations, sustainable innovative behaviors [26], and a new awareness of the value of gratitude to the different levels of relational life at the workplace, including decent work [27]. The gratitude framework could be promising for building healthy people, workers, organizations, and societies [23].

Regarding the measurement of gratitude, the theoretical structure of the construct of gratitude remains under discussion [28]. On the one side, gratitude is seen as a unitary concept and is thus explained by a unidimensional model (Gratitude Questionnaire-6, GQ-6) [5]. On the other side, it is considered as a non-unitary concept and thus represented by a multidimensional model with the following three dimensions: sense of abundance, appreciation of simple pleasure, appreciation of others (Gratitude Resentment and Appreciation Test, GRAT) [8]; Italian version Di Fabio [29] and of its short version (Gratitude Resentment and Appreciation Test-Revised Short, GRAT-RS) [30]. Hammer and Brenner [28] integrated the unidimensional and the multidimensional perspectives, advancing a bifactor model for the GRAT-RS where the three dimensions are also connected to a superordinate dimension of gratitude. This bifactor model for GRAT-RS was also confirmed for the Italian version of the scale [30]. Prior studies have evaluated the construct validity of the GRAT-RS using the classical theory test (CTT) [28,31]. Alternatively, to CCT, the Item Response Theory (IRT) [32] approach has been increasingly used. The IRT approach has the advantage of providing a nuanced item-based analysis, providing information on how well each item discriminates between different levels of an individual trait (e.g., between high and low gratitude). Moreover, the IRT perspective allows reliability estimation, again considering different levels of abilities [33]. In this framework, multidimensional IRT (MIRT) models were recently introduced [34]. MIRT models account for multiple latent traits in which each item is in a continuous multidimensional space [34]. Therefore, models can reflect more complex latent structures than unidimensional ones [34]. As a result, the MIRT approach could also yield novel results about GRAT-RS items, which are found to have the best fit for a bifactor latent structure [28,30]. Finally, MIRT is also a promising approach to realizing a fine-scale analysis of a multidimensional self-report tool according to accountability principles. These principles encourage researchers to apply evidence-based methods to guarantee the right balance between cost and effectiveness [35]. In organizations, the application of short-form questionnaires could be a valuable way to decrease the costs of research, safeguarding reliability [36].

The present study, therefore, aims at confirming and enriching analyses of the psychometric properties of the GRAT-RS—Italian version in workers by applying a MIRT approach.

## 2. Participants and Methods

### 2.1. Participants and Procedure

Five hundred and thirty-seven workers employed in public and private organizations from central Italy participated in the current study. They were 279 males (52%) and 258 females (48%) with a mean age of 45.1 (SD = 10.8). Participants were recruited via organizational gatekeepers. Workers participated voluntarily in the study. Written and informed consent was provided by the participants in the present study. This research adheres to privacy Italian laws (DL−196/2003) and EU 2016/679. The questionnaire was administered in paper-and-pencil format. Descriptive statistics of participants are provided in Table 1.

### 2.2. Measures

#### Gratitude Resentment and Appreciation Test-Revised Short (GRAT-RS)—Italian Version

The GRAT-RS [28,31]—Italian version [30] is a self-report scale composed of sixteen items with responses format ranging between 1 and 5 (Strongly disagree–Strongly agree, respectively). In line with the original English version, the GRAT-RS—Italian version [30] displayed a factor structure consistent with a bifactor solution composed of three specific factors and one general factor. In the Italian version, Cronbach’s alphas ranged between 0.91 and 0.83 [30]. Examples of items are as follows: for lack of a sense of deprivation [LOSD] factor “For some reason I don’t seem to get the advantages that others get”; for simple pleasures [SP] factor “I think it’s important to enjoy the simple things in life”; for social appreciation [SA] factor “I feel deeply appreciative for the things others have done for me in my life” [28,30]. The questionnaire includes some reversed items (the numbers 3, 6, 10, 11, 15). The mean scores of LOSD, SP, SA factors and the general factor are displayed in Table 1. The sixteen items of the GRAT-RS are shown in Table 3.

### 2.3. Data Analysis

R Studio for Windows (Version 2022.07.0, Posit Software, Boston, MA, USA) was applied to analyze the data. In each subsection, the specific R package employed for statistical analyses is provided.

#### 2.3.1. Confirmatory Factor Analysis

We carried out a confirmatory factor analysis (CFA) to confirm the GRAT-RS bifactor model founded by Hammer and Brenner [28] and Palazzeschi et al. [30]. Accordingly, four models were tested. The first model was a bifactor model (each item is simultaneously regressed on its respective factor [LOSD, SP, and SA] and onto a gratitude general factor [GEN]). The second was a correlational model (three factors: LOSD and SP with six items each; SA with four items). The third was a higher-order model (three factors: LOSD, SP, and SA regressed on a higher-order gratitude general factor [GEN]). The fourth model was a unidimensional model (item regressed onto a gratitude general factor [GEN]). The CFA was carried out by implementing the mean and variance-adjusted weighted least square estimation (WLSMV). Model fit was judged by means of the comparative fit index (CFI), the Tucker–Lewis Index (TLI), and the root mean square error of approximation (RMSEA). CFI and TLI values higher than 0.97 underlined a good fit; values ranging from 0.95 to 0.97 showed an acceptable fit. For RMSEA values were good (≤0.05), adequate (0.05–0.08), mediocre (0.08–0.10), and unacceptable (>0.10) [37]. The lavaan 0.6–9 and SemPlot 1.1.2 R packages (R Studio for Windows, Version 2022.07.0, Posit Software, Boston, MA, USA) were employed.

#### 2.3.2. Multidimensional Item Response Theory Analysis

In line with the bifactor measurement model of the GRAT-RS [28,30], a confirmatory multidimensional item response theory (MIRT) analysis was carried out [34]. MIRT is an IRT approach in which multiple underlying traits can be simultaneously measured [34,38]. Graded response model (GRM) [39] was used as a suitable bifactor MIRT model for the analysis of polytomous data [34]. In this model, two discrimination parameters can be calculated. The first discrimination parameter (a_1_) pertains to the general gratitude factor, whereas the others are one for each specific factor as follows: a_2_ refers to LOSD, a_3_ refers to SP, and a_4_ pertains to SA. Values < 0.64 reflect unacceptable discrimination, values 0.65–1.34 refer to moderate discrimination, values 1.35–1.69 point out high discrimination and values ≥1.70 reveal very high discrimination [40]. Furthermore, each item comprises four difficulty parameters (b_1_, b_2_, b_3_, b_4_) in line with the GRAT-RS 5-point Likert scale. Each threshold indicates the measured level of gratitude at which participants have a 50/50 chance of endorsing one or the other Likert scale option [39]. Item fit statistics were checked via the Orlando and Thissen [41] signed chi-squared test (S-X2) with quasi-Monte Carlo integration. S-X2 RMSEA values less than 0.05 indicates that an item fits the GRM MIRT model. In considering the bifactor structure of the GRAT-RS, Cai’s [42] Metropolis–Hastings Robbins–Monro algorithm was applied to estimate all parameters. The test information function (TIF) of general gratitude and specific factors were run to calculate reliability. The applied formula was 1 minus the inverse of the total information value [r = 1−(1/I)] [33]. TIF values > 3.30 (i.e., r = 0.70) indicated good reliability [43]. In this research, a sample size of *n* = 500 would be a robust estimation of item parameters (within 0.5 logits [contraction of log-odds probability units] at α = 0.05) with a minimum dropout of 15% [44]. The mirt 1.37.1 package (R Studio for Windows, Version 2022.07.0, Posit Software, Boston, MA, USA) was used.

#### 2.3.3. Reliability Analysis

According to the bifactor measurement model of the GRAT-RS [28,30], Cronbach’s alpha (α) and Omega (ω) coefficients [45] were calculated. Values of α and ω > 0.70 indicate good reliability [46,47]. The Psych 2.2.5 package (R Studio for Windows, Version 2022.07.0, Posit Software, Boston, MA, USA) was used.

## 3. Results

**Figure 1:**
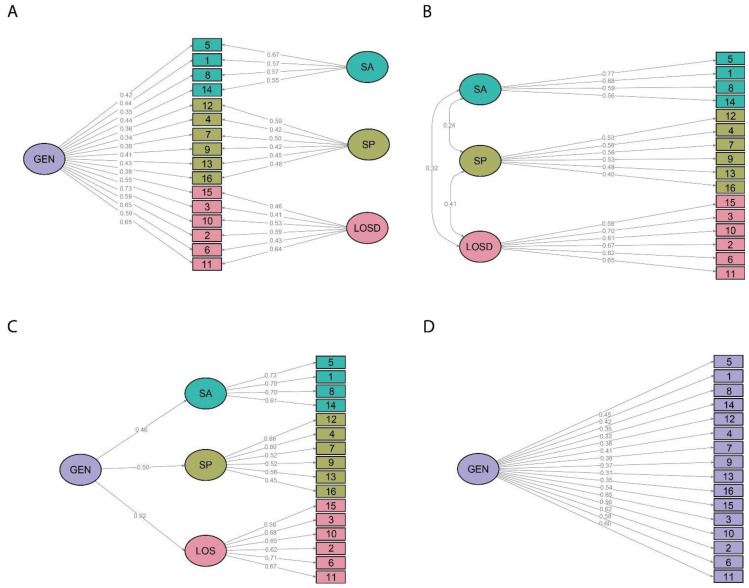
The Gratitude Resentment and Appreciation Test-Revised Short (GRAT-RS): factor analysis with weighted least squares means and variance adjusted (WLSMV) estimation. (**A**) bifactor model; (**B**) three-factor model; (**C**) higher-order model (**D**) unidimensional model (*n* = 537). GEN: general factor; LOSD: lack of a sense of deprivation specific factor; SP: simple pleasures specific factor; SA: social appreciation specific factor.

Table 2 shows the results of the confirmatory factor analyses, whereas Figure 1 shows the path diagrams of the four tested models. The bifactor model best fits the data (Table 1); thus, a bifactor multidimensional item response theory (MIRT) was selected to run the MIRT analysis.

Table 3 shows the factor loadings for the bifactor MIRT model of the GRAT-RS. Each item shows acceptable factor loadings ranging from 0.34 to 0.60 in the general gratitude factor and from 0.39 (LOSD) to 0.72 (SA) in the specific gratitude factors.

**Table 3:** The Gratitude Resentment and Appreciation Test-Revised Short (GRAT-RS): multidimensional item response theory (MIRT) analyses. Factor loadings of (*n* = 537).

	Bifactor			
	GEN	LOSD	SP	SA
GRAT-RS Item	λ	λ	λ	λ
2. Life has been good to me.	0.470	0.499		
3. There never seems to be enough to go around and I never seem to get my share.	0.573	0.495		
6. I really don’t think that I’ve gotten all the good things that I deserve in life.	0.603	0.387		
10. More bad things have happened to me in my life than I deserve.	0.489	0.497		
11. Because of what I’ve gone through in my life, I really feel like the world owes me something.	0.683	0.453		
15. For some reason I don’t seem to get the advantages that others get.	0.428	0.510		
4. Oftentimes I have been overwhelmed at the beauty of nature.	0.396		0.528	
7. Every Fall I really enjoy watching the leaves change colors.	0.346		0.507	
9. I think that it’s important to “Stop and smell the roses”.	0.405		0.390	
12. I think that it’s important to pause often to “count my blessings”.	0.379		0.405	
13. I think it’s important to enjoy the simple things in life.	0.373		0.619	
16. I think it’s important to appreciate each day that you are alive.	0.339		0.590	
1. I couldn’t have gotten where I am today without the help of many people.	0.341			0.562
5. Although I think it’s important to feel good about your accomplishments, I think that it’s also important to remember how others have contributed to my accomplishments.	0.337			0.611
8. Although I’m basically in control of my life, I can’t help but think about all those who have supported me and helped me along the way.	0.363			0.682
14. I feel deeply appreciative for the things others have done for me in my life.	0.339			0.718

GEN: general factor; LOSD: lack of a sense of deprivation specific factor; SP: simple pleasures specific factor; SA: social appreciation specific factor. λ: Factor loadings. Factor loadings > 0.30 are considered acceptable.

Table 4 shows the item parameter estimated via the MIRT-graded response model (GRM). All the items show a good fit to the GRM MIRT model (i.e., RMSEA ≤ 0.05). The discrimination parameters for the general and specific gratitude factors of the GRAT-RS were found from moderate to very high (Table 4). Furthermore, all the GRAT-RS items reported difficulty thresholds that proceeded from less to more difficulty, thus well reflecting the ordered categorical feature of the 5-point Likert scale (Table 4).

**Figure 2:**
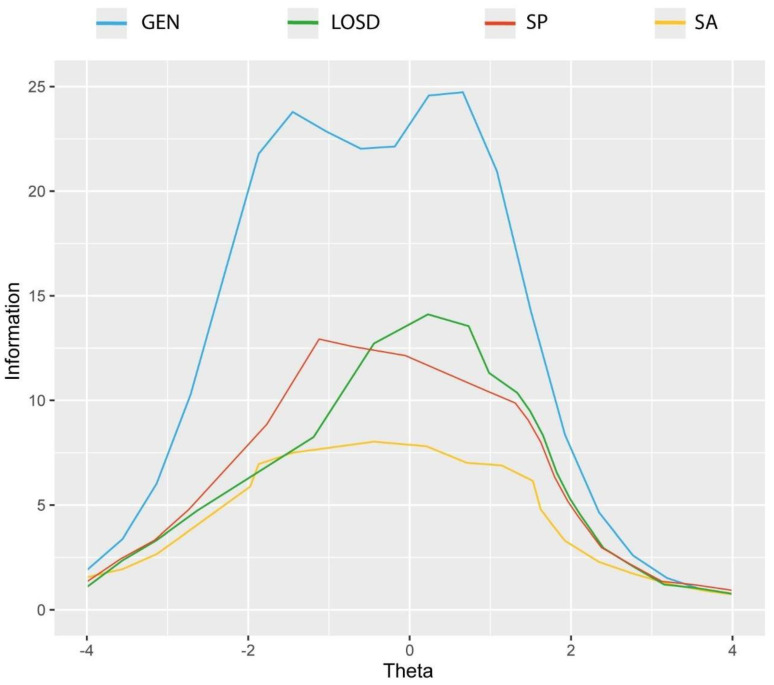
The Gratitude Resentment and Appreciation Test-Revised Short (GRAT-RS): Test information function (MIRT-grade response model) (*n* = 537). GEN: general factor; LOSD: lack of a sense of deprivation specific factor; SP: simple pleasures specific factor; SA: social appreciation specific factor.

Figure 2 reports the test information functions for the general gratitude factor (GEN) and the three specific gratitude factors (i.e., LOSD, SP, and SA).

The TIF curve of general gratitude factor (GEN) has its peak of information value at theta 1.81 (I = 24.91), with high reliability between θ = −3.74 (very low rigid gratitude) (I = 3.45; r = 0.71) and θ = 2.83 (high gratitude) (I = 3.75; r = 0.73) (Figure 2). LOSD factor showed the peak of information value at theta 0.62 (I = 13.78), with reliability ranging from θ = −3.14 to θ = 2.27 (Figure 2). SP factor showed the peak of information value at theta −1.12 (I = 12.52), with reliability ranging from θ = −3.16 to θ = 2.27 (Figure 2). Lastly, the SA factor showed the peak of information value at theta −0.53 (I = 7.66), with reliability ranging from θ = −2.83 to θ = 2.17 (Figure 2).

Table 5 shows GRAT-RS reliability indexes calculated according to the bifactor measurement model. General factor and all three subscales showed excellent reliability according to the Cronbach’s alpha (ranging from α = 0.80 [SP] to α = 0.89 [LOSD]) and McDonald’s Omega (ranging from ω = 0.91 [GEN] to ω = 0.89 [LOSD]) indexes (Table 5).

## 4. Discussion

The current research was aimed to enrich the understanding of the GRAT-RS—Italian version, exploring its psychometric properties in workers. To this end, a multidimensional item response theory (MIRT) analysis was run. The confirmatory factor analysis (CFA) showed that a bifactor solution had the best fit for the data. It is in line with previous research on Italian workers [30] and with the theoretical structure of the original scale [28]. Furthermore, the MIRT analysis confirmed the findings yielded via the CFA. The bifactor structure showed acceptable factor loading for the general and the specific GRAT-RS factors (i.e., LOSD, SP, and SA). Similarly, all the items fit well under the bifactor MIRT-GRM model, thus confirming the bifactor structure of the GRAT-RS.

The MIRT item-level statistics showed that each item has a suitable discrimination parameter, considering the general gratitude factor and the respective specific factors. Thus, GRAT-RS can precisely discriminate across different levels of gratitude, also considering each specific dimension of gratitude (i.e., LOSD, SP, and SA).

Finally, GRAT RS showed good MIRT reliability. In this line, GRAT general factors, LOSD, SP, and SA factors all had good reliability from low to high levels of the measured latent traits. Furthermore, CCT reliability indexes showed that the GRAT general factor as well as the LOSD, SP, and SA specific factors have good reliability, in line with previous studies [28,30].

## 5. Conclusions

The current study has limitations and strengths. The main limitation is that workers from central Italy were enrolled. Thus, results can only be generalized to some of the Italian contexts. However, the current study is the first study that has applied the MIRT approach to GRAT-RS and the homogeneity of participants, and sample size (i.e., >500 subjects) allowed an accurate MIRT parameter estimate [44]. Future research could also apply psychometric network analysis between GRAT-RS and the Big Five personality traits to deepen the study of the construct and gather new insights for research and intervention, as recently found in the literature for other crucial workplace variables [48]. Cross-cultural comparison is also recommended. Future research is also necessary to confirm the results from the MIRT perspective in workers from different groups (i.e., vulnerable workers, older workers, young workers and precarious workers). However, the reliable assessment of gratitude could be promising to detect gratitude in workplaces, which is a promising variable from a healthy organizational perspective focused on the promotion, development and maintenance of the well-being of workers and organizations for healthy business [23,49].

## Figures and Tables

**Table 1:** Mean age, gender of participants, and means participants’ scores obtained at the Gratitude Resentment and Appreciation Test-Revised Short (GRAT-RS) (*n* = 537).

Gender	*n* (%)
Male	279 (52)
Female	258 (48)
	M (SD)
Age	45.1 (10.8)
GRAT-RS	M (SD)
GEN	154.2 (18.8)
LOSD	63.0 (11.1)
SP	53.3 (8.2)
SA	37.9 (5.9)

GEN: general factor; LOSD: lack of a sense of deprivation specific factor; SP: simple pleasures specific factor; SA: social appreciation specific factor.

**Table 2:** The Gratitude Resentment and Appreciation Test-Revised Short (GRAT-RS): confirmatory factor analysis with weighted least squares means and variance adjusted (WLSMV) estimation. Fit indexes (*n* = 537).

Models	χ^2^(df)	CFI	TLI	RMSEA [95% CI]
Bifactor	161.573 (88)	0.980	0.973	0.039 [0.30–0.49]
Correlational	302.369 (101)	0.945	0.935	0.061 [0.053–0.069]
Higher order	302.396 (101)	0.945	0.935	0.061 [0.053–0.069]
Unidimensional	741.719 (104)	0.827	0.801	0.107 [0.100–0.114]

**Table 4:** The Gratitude Resentment and Appreciation Test-Revised Short (GRAT-RS): multidimensional item response theory (MIRT) analyses. Discrimination and difficulties parameters (grade response model) (*n* = 537).

GRAT-RS Item	a_1_	a_2_	a_3_	a_4_	b_1_	b_2_	b_3_	b_4_	RMSEA
Item 2	1.10	1.17	0.00	0.00	−1.98	−0.39	2.33	4.40	0.008
Item 3	1.49	1.29	0.00	0.00	−1.85	−0.76	2.27	4.70	0.013
Item 6	1.47	0.94	0.00	0.00	−1.73	−0.42	2.71	4.91	0.000
Item 10	1.16	1.18	0.00	0.00	−2.20	−0.78	2.50	4.88	0.015
Item 11	2.03	1.34	0.00	0.00	−2.10	−0.54	2.49	5.68	0.026
Item 15	0.98	1.16	0.00	0.00	−2.00	−1.09	2.16	4.70	0.030
Item 4	0.68	0.00	1.13	0.00	−3.37	−0.96	2.95	4.58	0.035
Item 7	0.75	0.00	1.09	0.00	−2.48	−0.64	3.37	4.62	0.033
Item 9	0.83	0.00	0.80	0.00	−2.78	−1.12	2.62	4.60	0.000
Item 12	0.77	0.00	0.83	0.00	−2.14	−1.27	2.41	4.14	0.034
Item 13	0.92	0.00	1.53	0.00	−3.25	−0.74	3.47	5.07	0.026
Item 16	0.79	0.00	1.37	0.00	−1.69	−0.40	2.94	5.23	0.023
Item 1	0.77	0.00	0.00	1.27	−2.39	−0.04	2.07	3.92	0.000
Item 5	0.80	0.00	0.00	1.45	−2.17	−0.02	1.60	3.82	0.000
Item 8	0.97	0.00	0.00	1.83	−2.15	−0.15	1.93	4.32	0.005
Item 14	0.95	0.00	0.00	2.01	−3.03	−0.48	1.51	4.22	0.000

a_1_ = discrimination parameter for general factor (GEN); a_2_ = discrimination parameter for lack of a sense of deprivation specific factor (LOSD); a_3_ = discrimination parameters for simple pleasures specific factor (SP); a_4_ = discrimination parameters for social appreciation specific factor (SA). b_1_, b_2_, b_3_, b_4_ = difficulties parameters. RMSEA: signed chi-squared test root mean squared error of approximation.

**Table 5:** The Gratitude Resentment and Appreciation Test-Revised Short (GRAT-RS): indexes of reliability (*n* = 537).

Reliability	GEN	LOSD	SP	SA
Cronbach’s Alpha (a)	0.85	0.89	0.80	0.87
Omega (ω)	0.91	0.89	0.83	0.87

GEN: general factor; LOSD: lack of a sense of deprivation specific factor; SP: simple pleasures specific factor; SA: social appreciation specific factor.

## Data Availability

The data presented in this study are available from the corresponding author on reasonable request. The data are not publicly available due to privacy reasons.

## References

[1] Emmons R.A., Shelton C.M., Snyder C.R., Lopez S.J. (2002). Gratitude and the science of positive psychology. Handbook of Positive Psychology.

[2] Pruyser P.W. (1976). The Minister as Diagnostician: Personal Problems in Pastoral Perspective.

[3] Lazarus R.S. (1991). Emotion and Adaptation.

[4] Lazarus R.S., Lazarus B.N. (1994). Passion and Reason: Making Sense of Our Emotions.

[5] McCullough M.E., Emmons R.A., Tsang J.A. (2002). The grateful disposition: A conceptual and empirical topography. J. Pers. Soc. Psychol..

[6] Wood A.M., Froh J.J., Geraghty A.W. (2010). Gratitude and well-being: A review and theoretical integration. Clin. Psychol. Rev..

[7] Weiner B. (1986). An Attributional Theory of Motivation and Emotion.

[8] Watkins P.C., Woodward K., Stone T., Kolts R.L. (2003). Gratitude and happiness: Development of a measure of gratitude, and relationships with subjective well-being. Soc. Behav. Pers..

[9] Seligman M.E.P., Snyder C.R., Lopez S.J. (2002). Positive psychology, positive prevention, and positive therapy. Handbook of Positive Psychology.

[10] Seligman M.E.P., Csikszentmihalyi M. (2000). Positive psychology: An introduction. Am. Psychol..

[11] Emmons R.A., McCullough M.E. (2003). Counting blessings versus burdens: An experimental investigation of gratitude and subjective well-being in daily life. J. Pers. Soc. Psychol..

[12] Lyubomirsky S., Dickerhoof R., Boehm J.K., Sheldon K.M. (2011). Becoming happier takes both a will and a proper way: An experimental longitudinal intervention to boost well-being. Emotion.

[13] Rash J.A., Matsuba M.K., Prkachin K.M. (2011). Gratitude and well-being: Who benefits the most from a gratitude intervention?. Appl. Psychol..

[14] Di Fabio A., Saklofske D.H. (2021). The relationship of compassion and self-compassion with personality and emotional intelligence. PAID 40th anniversary special issue. Personal. Individ. Differ..

[15] Hage S.M., Romano J.L., Conyne R.K., Kenny M., Matthews C., Schwartz J.P., Waldo M. (2007). Best practice guidelines on prevention practice, research, training, and social advocacy for psychologists. Couns. Psychol..

[16] Sawyer K.B., Thoroughgood C.N., Stillwell E.E., Duffy M.K., Scott K.L., Adair E.A. (2002). Being present and thankful: A multi-study investigation of mindfulness, gratitude, and employee helping behavior. J. Appl. Psychol..

[17] Siu O.L., Cooper C.L., Phillips D.R. (2014). Intervention studies on enhancing work well-being, reducing burnout, and improving recovery experiences among Hong Kong health care workers and teachers. Int. J. Stress Manag..

[18] Emmons R.A., Cameron K.S., Dutton J.E., Quinn R.E. (2003). Acts of gratitude in organizations. Positive Organizational Scholarship: Foundations of a New Discipline.

[19] Loi N.M., Ng D.H. (2021). The relationship between.gratitude, wellbeing, spirituality, and experiencing meaningful work. Psych.

[20] Di Fabio A., Blustein D.L. (2016). From Meaning of Working to Meaningful Lives: The Challenges of Expanding Decent Work. Front. Psychol. Organ. Psychol..

[21] Svicher A., Gori A., Di Fabio A. (2022). Work as Meaning Inventory: A network analysis in Italian workers and students. Aust. J. Career Dev..

[22] Di Fabio A., Palazzeschi L., Bucci O. (2017). Gratitude in organizations: A contribution for healthy organizational contexts. Front. Psychol. Organ. Psychol..

[23] Di Fabio A., Cheung F., Peiró J.-M. (2020). Editorial Special Issue Personality and individual differences and healthy organizations. Personal. Individ. Differ..

[24] Avolio B.J., Gardner W.L. (2005). Authentic leadership development: Getting to the root of positive forms of leadership. Leadersh. Q..

[25] Di Fabio A., Peiró J.M. (2018). Human Capital Sustainability Leadership to promote sustainable development and healthy organizations: A new scale. Sustainability.

[26] Duradoni M., Di Fabio A. (2019). Intrapreneurial Self-Capital and Sustainable Innovative Behavior within Organizations. Sustainability.

[27] Di Fabio A., Svicher A., Gori A. (2021). Occupational Fatigue: Relationship with Personality Traits and Decent Work. Front. Psychol. Organ. Psychol..

[28] Hammer J.H., Brenner R.E. (2019). Disentangling gratitude: A theoretical and psychometric examination of the gratitude resentment and appreciation test–revised short (GRAT–RS). J. Pers. Assess..

[29] Di Fabio A. (2016). Gratitude Resentment and Appreciation Test: Primo contributo alla validazione della versione italiana [Gratitude Resentment and Appreciation Test: First contribution to the validation of the Italian version]. Counseling.

[30] Palazzeschi A., Svicher A., Gori A., Di Fabio A. (2022). Gratitude in organizations: Psychometric properties of the Italian version of the Gratitude Resentment and Appreciation Test–Revised Short (GRAT–RS) in Workers. Int. J. Environ. Res. Public Health.

[31] Thomas M., Watkins P. Measuring the grateful trait: Development of revised GRAT. Proceedings of the Annual Convention of the Western Psychological Association.

[32] Van der Linden W.J. (2017). Handbook of Item Response Theory.

[33] Embretson S.E., Reise S.P. (2000). Item Response Theory for Psychologists.

[34] Reckase M.D. (2009). Multidimensional Item Response Theory Models. Multidimensional Item Response Theory. Statistics for Social and Behavioral Sciences.

[35] Whiston S.C. (1996). Accountability Through Action Research: Research Methods for Practitioners. J. Couns. Dev..

[36] Whiston S.C. (2001). Selecting career outcome assessments: An organizational scheme. J. Career Assess..

[37] Schermelleh-Engel K., Moosbrugger H., Müller H. (2003). Evaluating the Fit of Structural Equation Models: Tests of Significance and Descriptive Goodness-of-Fit Measures. Methods Psychol. Res..

[38] Bock R.D., Aitkin M. (1981). Marginal maximum likelihood estimation of item parameters: An application of the EM algorithm. Psychometrika.

[39] Samejima F. (1968). Estimation of latent ability using a response pattern of graded scores. ETS Res. Bull. Ser..

[40] Baker F.B. (2001). The Basics of Item Response Theory.

[41] Orlando M., Thissen D. (2000). Likelihood-based item-fit indices for dichotomous item response theory models. Appl. Psychol. Meas..

[42] Cai L. (2010). Metropolis-Hastings Robbins-Monro algorithm for confirmatory item factor analysis. J. Educ. Behav. Stat..

[43] Cappelleri J.C., Jason Lundy J., Hays R.D. (2014). Overview of Classical Test Theory and Item Response Theory for the Quantitative Assessment of Items in Developing Patient-Reported Outcomes Measures. Clin. Ther..

[44] Linacre J.M. (1994). Sample size and item calibration stability. Transformation.

[45] McDonald R.P. (1999). Test Theory: A Unified Approach.

[46] Nunnally J.C., Bernstein I.H. (1994). Psychometric Theory.

[47] Adams R.J. (2005). Reliability as a measurement design effect. Stud. Educ. Eval..

[48] Di Fabio A., Saklofske D.H., Gori A., Svicher A. (2022). Perfectionism: A network analysis of relationships between the Big Three dimensions and the Big Five Personality traits. Personal. Individ. Differ..

[49] Johnson S., Robertson I., Cooper C.L. (2018). Wellbeing: Productivity and Happiness at Work.

